# Role of Dietary Protein and Muscular Fitness on Longevity and Aging

**DOI:** 10.14336/AD.2017.0202

**Published:** 2018-02-01

**Authors:** Barbara Strasser, Konstantinos Volaklis, Dietmar Fuchs, Martin Burtscher

**Affiliations:** ^1^Division of Medical Biochemistry, Biocenter, Medical University Innsbruck, Austria; ^2^Department of Prevention and Sports Medicine, TUM, Munich, Germany; ^3^Division of Biological Chemistry, Biocenter, Medical University Innsbruck, Austria; ^4^Department of Sport Science, Medical Section, University Innsbruck, Austria

**Keywords:** Aging, muscle, physical fitness, protein, kynurenine pathway, longevity

## Abstract

Muscle atrophy is an unfortunate effect of aging and many diseases and can compromise physical function and impair vital metabolic processes. Low levels of muscular fitness together with insufficient dietary intake are major risk factors for illness and mortality from all causes. Ultimately, muscle wasting contributes significantly to weakness, disability, increased hospitalization, immobility, and loss of independence. However, the extent of muscle wasting differs greatly between individuals due to differences in the aging process *per se* as well as physical activity levels. Interventions for sarcopenia include exercise and nutrition because both have a positive impact on protein anabolism but also enhance other aspects that contribute to well-being in sarcopenic older adults, such as physical function, quality of life, and anti-inflammatory state. The process of aging is accompanied by chronic immune activation, and sarcopenia may represent a consequence of a counter-regulatory strategy of the immune system. Thereby, the kynurenine pathway is induced, and elevation in the ratio of kynurenine to tryptophan concentrations, which estimates the tryptophan breakdown rate, is often linked with inflammatory conditions and neuropsychiatric symptoms. A combined exercise program consisting of both resistance-type and endurance-type exercise may best help to ameliorate the loss of skeletal muscle mass and function, to prevent muscle aging comorbidities, and to improve physical performance and quality of life. In addition, the use of dietary protein supplementation can further augment protein anabolism but can also contribute to a more active lifestyle, thereby supporting well-being and active aging in the older population.

It is well known that people of all ages benefit from regular physical activity, which reduces the risk of coronary heart disease, hypertension, certain kinds of cancer, type 2 diabetes, and many other chronic diseases. Indeed, a low level of cardiorespiratory fitness is accepted nowadays as a powerful predictor of mortality in healthy as well as diseased individuals [1-3]. Muscular strength is an important component of physical fitness with an independent role in the prevention of many chronic diseases. Several epidemiological studies have shown that muscular weakness in middle-aged and older individuals is strongly related to functional limitations and physical disability [4-6]. Furthermore, epidemiological or short-term studies indicate a potential beneficial effect of increasing protein intake in older adults. Thus, the main goal of the present paper is to provide an overview on the role of physical exercise in muscle health in old age and to outline the clinical evidence of dietary protein intake to support healthy aging.

## Muscular strength and longevity

A growing body of evidence suggests that muscular strength is inversely and independently associated with all-cause and cardiovascular mortality even after adjusting for cardiorespiratory fitness and other cofactors such as age, body fat, and smoking [7-12]. Several studies have shown that muscular strength is inversely associated with the incidence of many chronic diseases such as cardiovascular disease and stroke [13-17], hypertension [18], metabolic syndrome or hyperinsulinemia [19, 20], and type 2 diabetes [21]. In a large cohort study of one million Swedish men, muscle strength in young adulthood was an important predictor of coronary heart disease and stroke risk in later life, and this association persisted for both normal weight and obese individuals [14]. Recently, some researchers have also tried to relate muscular strength to the risk of suffering from multiple chronic diseases. In a cross-sectional study, including 1,145 subjects aged 50 years and older, Cheung *et al*. [22] found that handgrip strength in men was a more useful marker of multimorbidity than chronological age. Results from the KORA-Age study, a population-based study of 1,079 older people, demonstrated that low grip strength is inversely and independently associated with multimorbidity among older women after controlling for traditional confounders, as well as for inflammatory markers, telomere length, and levels of physical activity [23]. In addition, studies among older people have further suggested that there is a strong association between low muscle strength and both cognitive impairments and the risk of neurodegenerative diseases, such as dementia, Alzheimer’s disease, and Parkinson disease [24-28]. All the above facts are of great interest from a public health perspective as muscular strength is a modifiable risk factor that can substantially influence chronic disease risk and premature mortality.

Multiple studies have examined the association between muscular strength and all-cause mortality, and all reported significant mortality reductions with increased levels of muscular strength [29-35]. This strong association persisted after adjusting for several cofactors and comorbidities and even after controlling for cardiorespiratory fitness. Especially in the oldest old population, poor handgrip strength has been linked with premature mortality, and this association tended to be stronger in women [36]. According to the findings of the Leiden 85-plus study [37], a population-based study that involved all (n=555) 85-year-old inhabitants of Leiden (The Netherlands), the risk of all-cause mortality was elevated by 35% and 104% in the lowest tertiles of handgrip strength among participants at age 85 and 89 years respectively. Interestingly, in this study, it was also shown that handgrip strength had a greater impact on mortality than the age of the participants. Two other studies clearly confirmed the strong association between muscular strength and mortality in older as well as in younger populations. A meta-analysis of 53,476 older individuals found that the hazard ratio for all-cause mortality comparing the weakest with the strongest quintile of grip strength was 1.67 after adjustment for age, gender, and body size [38]. In the Prospective Urban-Rural Epidemiology (PURE) study, Leong *et al*. [11] followed 139,691 adults aged between 35 and 70 years living in 17 countries for a median time of 4 years in order to assess the prognostic value of grip strength on mortality. They demonstrated that grip strength was inversely associated with all-cause mortality, cardiovascular mortality, non-cardiovascular mortality, myocardial infarction, and stroke.

## Biology of muscle aging

Human skeletal muscle inevitably undergoes remarkable changes with aging, characterized by a decline in muscle mass and strength of about 1% per year from the age of around 40 years [39]. Ultimately, muscle wasting will contribute significantly to frailty, immobility, and loss of independence. However, the extent of muscle wasting differs greatly between individuals due to differences in the aging process *per se* as well as in physical activity levels. Alterations in muscle architecture and fiber type composition, in tendon mechanical properties and vascular control of the contracting muscle are the most prominent characteristics associated with the decline in mass and functioning of aging skeletal muscle [40-42].

### Age-related changes in muscle architecture

Distinct alterations in muscle architecture occur during aging resulting from inactivity (disuse atrophy) and originating from the aging process (senile sarcopenia) [42]. Whereas disuse atrophy is characterized only by a reduction in fiber size, sarcopenia exhibits both reduced fiber size and reduced fiber number. In addition, fascicle length and pennation angle decrease with aging [43]. The observation that the physiological cross-sectional area (muscle volume divided by fascicle length) declines with disuse and aging as well indicates a more pronounced loss of sarcomeres in parallel than sarcomeres in series (fascicle length) [42].

### Age-related changes in fiber type composition

Reduced muscle volume in the elderly results from reductions in motor units and muscle fibers. Whereas the size of type 1 muscle fibers is nearly maintained, type 2 fiber size diminishes [44]. Nevertheless, the loss of fibers remains the main reason for the reduced muscle mass and strength with aging. Type 2 fibers seem to be particularly prone to increasing denervation with increasing age [45]. Fiber loss, however, is at least partly prevented because type 1 motor neurons form connections to denervated type 2 fibers. As a consequence, type 1 motor neurons become enlarged at the expense of type 2 fibers [45]. Muscle unloading (disuse) provokes a slow-to-fast transition, indicated by the elevation of fast myosin heavy chain (MHC) isoforms as well as fast myosin light chain (MLC) isoforms [46]. In contrast to disuse, aging *per se* results in a fast-to-slow transition, partly explained by the denervation of type 2 fibers and the glycation of the MHC [47].

**Figure 1.**
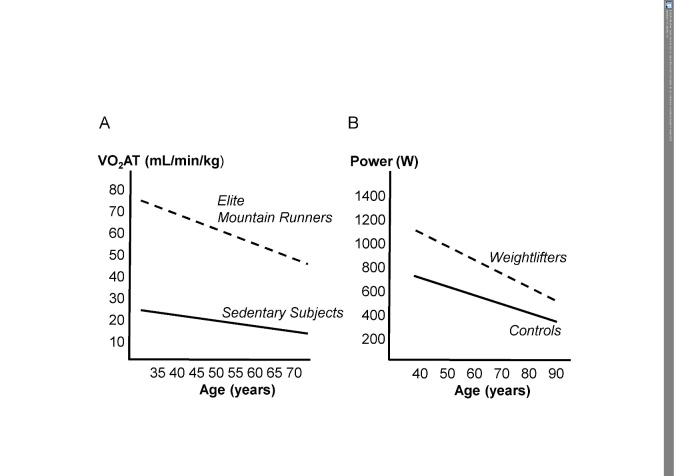
Age-related decline of endurance and strength performance in trained versus untrained subjects Age-related decline in the anaerobic threshold (VO_2_AT) in Master’s mountain runners and sedentary subjects (A) and peak power in Master’s weight lifters and sedentary control subjects (B); (modified from ref. 58, 59).

### Mechanisms proposed

Several mechanisms besides physical inactivity may explain aging-related muscle wasting. Age-related changes in the cerebral cortex have been proposed as a potential contributing factor, although no significant loss of motor cortical neurons seems to occur there [48]. However, there is a progressive loss of motor units (MUs) during the first five or six decades of life, which accelerates thereafter [49]. Axonal atrophy with aging may result from reduced axonal transport, degeneration of mitochondria, and accumulations of filaments also seen in neurodegenerative diseases [50]. Consequently, a denervation of type 2 fibers and their reinnervation by type 1 MUs probably develops in relation to the alterations in testosterone and estrogen (explaining sex differences), thyroid hormone levels, mitochondrial dysfunction, oxidative stress, low-grade inflammation, and insulin resistance [51]. In particular, the functional deterioration of mitochondria with aging and the related increase in oxidative stress may represent an important pathophysiological process of aging [52]. In addition, vasodilation and vascular control seem to be compromised in the aging skeletal muscle contributing to the decline in exercise performance. Responsible mechanisms may include smaller and stiffer vessels, impaired endothelium-dependent vasodilation, increased sympathetic vasoconstriction, alterations in metabolic or myogenic control, and diminished effectiveness of the skeletal muscle pump [42]. Most importantly, lifestyle characteristics such as physical activity and dietary habits have the potential to modify the aging process significantly.

## Physiological response to exercise training

The rapid and pronounced effects of resistance training (concentric and/or eccentric) on strength and muscle mass in healthy adults are as well established as the beneficial implications of endurance exercise on the cardiovascular and skeletal muscle systems. Mitochondrial biogenesis promoted by exercise (endurance and/or strength) contributes significantly to the beneficial outcomes of exercise training [53]. There is also convincing evidence that resistance training of sufficient intensity is an effective measure for counteracting muscle wasting and frailty in very old people [54]. Apart from the aging process *per se*, disuse often contributes importantly to muscle wasting and decline in cardiovascular fitness. Examples of dramatic disuse-related reductions in strength and muscle mass are bed rest and weightlessness [55]. In contrast, acute resistance exercise enhances myofibrillar muscle protein synthesis in young as well as in older individuals of both sexes [56]. Thus, physical inactivity promotes a circulus vitiosus in aging individuals, leading to coordinated deadaptation of the cardiovascular and skeletal muscle systems, which are in charge of oxygen delivery to and oxygen utilization in working muscles [57]. In contrast, exercise training counteracts this circulus vitiosus, resulting in coordinated beneficial adaptations of these systems [57]. The much higher physical fitness (strength and endurance) levels of aging athletes compared with their sedentary peers are largely explained by their regular high-intensity physical activity throughout the life span [58, 59] (Fig. 1).

### Resistance type of exercise

A single bout of resistance training is associated with a two- to threefold increase in muscle protein synthesis, which may be additionally enhanced by the intake of a protein-rich diet [60, 61]. Elderly sedentary subjects can achieve up to more than 50% strength gain even after 6 weeks of resistance training when performing two or three sessions per week applying a sufficiently high intensity (about 70-80% of maximal strength) [62]. To cause essential hypertrophy of muscle fibers, additional new myonuclei are necessary [63]. Skeletal muscle fibers are multinucleated, and these myonuclei are post-mitotic and cannot proliferate. Thus, for repair and renewal of myofibers, myogenic progenitor cells, termed satellite cells, are competent [64]. These satellite cells are in a quiescent state and only become activated when exposed to stress such as weight bearing or trauma resulting in proliferation and differentiation into new myonuclei, which fuse with existing muscle fibers [64]. It is believed that the increase in the number of satellite cells in response to various types of resistance training is different between young and older individuals, likely representing a limiting factor for muscle fiber hypertrophy in the elderly [63]. With regard to gender, resistance training has been shown to increase the myonuclear and satellite cell contents of type II muscle fibers and related muscle mass with no differences between older men and women [65]. Finally, resistance training also has the potential to increase respiratory capacity and the intrinsic function of mitochondria of skeletal muscles [66].

### Endurance type of exercise

Endurance exercise training is the method of choice to maintain or improve cardiovascular fitness. Aerobic capacity (VO_2_max) has been shown to improve greatly in young as well as older (about 70 years) subjects in response to 12 weeks of endurance training [67]. There was a linear increase in VO_2_max during the 12-week training period, finally amounting to 30% improvement in the older group. Although this VO_2_max increase was mainly achieved (about 70%) by an increase in maximal cardiac output, oxygen extraction in working muscles significantly contributed to the improvement in aerobic capacity [67]. These training adaptations occurring in skeletal muscle contribute importantly to the ability to perform sustained exercise [57]. Muscle adaptations to repeated bouts of endurance exercise include increased capillary supply, elevations in key enzyme activities of the mitochondrial electron transport chain, and related enhancement in mitochondrial protein accumulation [57]. As a consequence, trained muscles at the same exercise intensity show a higher rate of fat oxidation, less use of muscle glycogen, and less lactate production, all contributing to improved exercise tolerance. Endurance exercise interventions have been reported to increase mitochondrial content on account of upregulation of transcriptional regulators of mitochondrial biogenesis [68]. The authors observed marked increases in the gene expression of peroxisome proliferator-activated receptor-γ coactivator-1 (PGC-1α) by 50% and of mitochondrial transcription factor A (TFAM) comparable to that found in older subjects after a similar exercise intervention [69]. Taken together, a combined exercise program consisting of both resistance-type and endurance-type exercise may best help to ameliorate the loss in skeletal muscle mass and function, prevent muscle aging comorbidities, and improve physical performance and quality of life.

## Dietary protein, muscle, and healthy aging

Protein nutrition is an important component of the diet of older individuals. Protein is an essential nutrient; thus, at least a minimal amount of protein intake is necessary to support healthy living. However, older individuals are at high risk of insufficient protein intake, most probably as a consequence of aging malnutrition and anabolic resistance in aged muscle [70]. Furthermore, concomitant inflammation observed in chronic diseases leads to protein degradation and reduced skeletal muscle protein synthesis (MPS) and, consequently, to higher protein requirements [71]. Therefore, the current recommended dietary allowance (RDA) for protein of 0.8 grams of protein per kilogram of body mass per day might not be adequate for maintaining muscle and bone health in old age [72]. Recent research has provided evidence of the additional benefits of a greater dietary protein intake (i.e., 1.5 g/kg body mass/day) beyond the prevention of sarcopenia [73], most relevant in both the genesis of and recovery from fractures [74, 75]. Still, one of the major issues regarding protein intake is identifying how proteins derived from animal and plant sources differ in their capacity to enhance immunity in elderly people and how much protein is needed as the combination of exercise and protein ingestion has a positive, often synergistic effect on MPS [76-78].

### Anabolic resistance of MPS with aging—the importance of exercise

With aging, a progressive loss of skeletal muscle mass (defined as sarcopenia) occurs at a rate of 3-8% each decade after the age of 30 years [79], which has been attributed to impaired skeletal MPS, triggered by reduced amino acid delivery to aged skeletal muscle [80]. Older people appear to have a blunted muscle protein synthetic response to the two main anabolic stimuli, protein administration [81] or resistance exercise [82]. Several factors may influence the stimulation of MPS after a protein meal that may lead to anabolic resistance with aging. These include impairments in protein digestion and amino acid absorption [83], postprandial muscle tissue perfusion [84], muscle uptake of dietary amino acids [85], or a reduced amount or activation of anabolic signaling proteins [81, 86]. However, Burd and colleagues highlighted the hypothesis that physical inactivity is a key factor responsible for the proposed anabolic resistance of MPS with aging [87]. Indeed, several studies have shown that physical exercise performed before protein intake augments muscle protein synthetic response to protein ingestion and allows more of the ingested protein-derived amino acids to be used for *de novo* MPS in aging muscle [78, 88]. A recent study found that older individuals who are perhaps unable to consume large amounts of protein can still benefit from ingesting smaller amounts of protein before sleep by performing exercise beforehand, thereby increasing the overnight muscle protein synthetic response [76]. This simple strategy may help to preserve muscle mass and strength in the older population and, as such, support healthy aging.

### Clinical benefits of protein supplementation

Muscle atrophy is an unfortunate effect of aging and many diseases and can compromise physical function and impair vital metabolic processes [89]. Interventions for sarcopenia include exercise and nutrition [90, 91], because both have a positive impact on protein anabolism but also enhance other aspects that contribute to well-being in sarcopenic older adults, such as physical function, quality of life, and anti-inflammatory state [92]. Resistance training leads to a genuine increase in lean body mass and muscle strength in healthy older adults and is therefore considered to be the best exercise method for the treatment of sarcopenia [93]. Growing evidence supports increasing muscle protein uptake through nutrient interventions coupled with appropriate contractile manipulation [94]. Of importance for older adults, low load weight lifting effectively stimulates the rates of MPS to a level comparable with traditional high loads, besides other benefits such as improved aerobic capacity [95].

In many wasting diseases, muscle atrophy can be attributed to detrimental metabolic changes inducing catabolic crises. For example, rapid muscle wasting occurs early in critical illness, the extent of which determines recovery and survival [96, 97]. Thus, early interventions to enhance anabolism are required. Functional electrical stimulation has become a clinically established method to prevent the loss of muscle mass for patients who are not able to perform active exercise [98]. This technique appears to be a useful adjunct to reverse muscle wasting in long-term intensive care unit patients by reducing protein degradation and inflammation in postoperative patients, which can positively affect the immune and inflammatory response seen in critical illness [99]. Furthermore, protein energy malnutrition is a condition that affects many hospital patients and consists of a variety of alterations including decreased intake of calories and/or protein and excess weight loss [100]. Insufficient dietary intake is not only related to the development of sarcopenia [101], but is also a major risk factor for illness and mortality in older hospitalized medical patients [102]. In malnourished older patients, short-term protein supplementation solely significantly increased both dietary intake and lean body mass [103]. Furthermore, immunonutrition has become a popular approach to augment the immune response of medically ill, immobilized patients. However, in critical medical care settings, nourishment alone has not improved clinical outcomes in numerous controlled trials [104, 105]. Indeed, the results of a recent systematic review of randomized clinical trials demonstrates that, although nutritional interventions increase daily caloric and protein intake as well as body weight, there is little effect of nutritional support on clinical outcomes in malnourished medical inpatients [106]. Perhaps that aggressive nutrition might not be sufficient unless you train these patients, because low skeletal muscle area may play a significant role. Muscularity represents a potential new marker for identifying mortality risk but, more importantly, permits the early identification of patients who may benefit from integrated immune-modulating nutrition. High-quality randomized clinical trials are needed to fill this gap.

The aging muscle is also a significant predictor of falls and fractures associated with a loss of independence in old age [107, 108]. Thus, anabolic interventions against sarcopenia are particularly relevant in this cohort, as it is more prevalent in older hip fracture patients [109]. Although protein energy undernutrition predicts poor outcome in hip fracture patients, increased energy and protein intake have a favorable effect on the postoperative course in older individuals with hip fractures [110]. In summary, clinicians should not overlook the benefit of combined exercise and protein ingestion. Muscle stimulation is essential in order to prevent muscle wasting, maintain normal muscle function, and reduce inflammation in hospitalized patients; these are crucial ways to attenuate infection development and mortality. In addition, there is a clear need for dietary protein intake above the current RDA in older individuals, especially during periods when musculoskeletal mass is compromised, such as immobilization, with experts recommending between 1.2 and 2.0 g/kg body mass per day [111].

### Protein quantity, quality, and timing of consumption

Several factors related to protein nutrition, including the dose, source, and timing of ingested protein, as well as the co-ingestion of other macronutrients may influence the magnitude of the muscle protein response to exercise. It is clear that the essential amino acids are critical for optimal stimulation of MPS. It is generally accepted that the optimal amount of protein ingestion following exercise to stimulate maximal rates of MPS is ≈20-25g [112]. However, recent data suggest that this amount may be insufficient in the elderly [113]. Stimulation of MPS in older adults increases, even up to 40 g of protein intake during recovery from resistance-type exercise. Yet, consuming large protein quantities in a single meal may be difficult for older people [114]. To solve this problem, experts recommend the ingestion of “suboptimal” doses of protein via supplementation with specific amino acids such as leucine [115-117]. Leucine is a powerful signal for stimulation of the mammalian target of the rapamycin complex-1 (mTORC1) pathway, which is responsible for the initiation of protein translation and is thus often used as a proxy measure for MPS [118]. Animal proteins have a higher proportion of the amino acid leucine. Whey protein is most effective in stimulating post-exercise MPS when compared with casein or soy protein [119]. The timing of protein ingestion represents another important factor for muscle protein anabolism. Although isolated proteins (e.g., whey, soy) should be consumed during or immediately after an exercise bout, the ingestion of protein-dense foods, such as dairy and meat, should be 90-120 minutes prior to exercise [91]. Most important for older adults, however, is to consume an adequate amount of high-quality protein at each meal, in combination with physical exercise.

## Tryptophan-kynurenine metabolism and immune activation in aging

**Figure 2.**
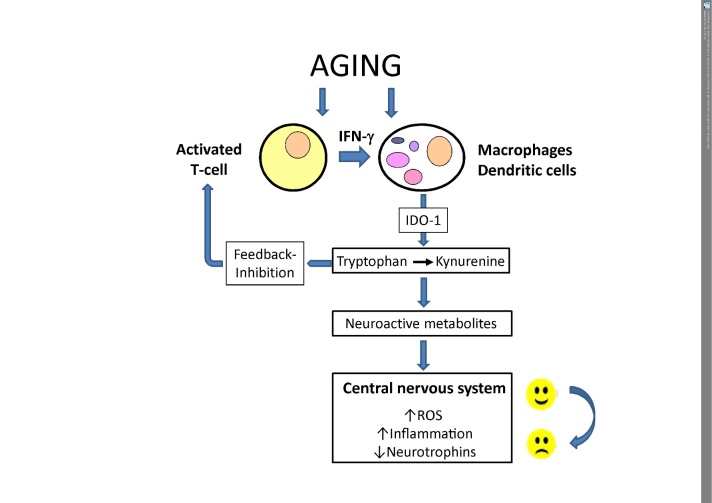
The induction of indoleamine 2,3-dioxygenase 1 (IDO1) by aging The process of aging involves pro-inflammatory pathways which include activation of the T-cell-macrophage axis in the framework of the cell-mediated (Th1-type) immune response in which the formation of Th1-type cytokine interferon-γ (IFN-γ) is of utmost relevance. IFN-γ stimulates a broad spectrum of biochemical pathways that are directed to stop unwanted growth of pathogens or malignant cells. Among them, the conversion of essential amino acid tryptophan to kynurenine is a key element, which on the one hand is involved in a feedback inhibition of T-cell activation via regulatory T-cells and thus immunosuppressive. On the other hand, the catabolites generated by this strategy can impact on the central nervous system when neuroactive compounds accumulate and pro-inflammatory cascades including the formation of reactive oxygen species (ROS) interfere with neuroendocrine signaling, which controls mood and behavior.

Immune activation in aging influences the metabolism of amino acids [120]. Although less than 1% of dietary tryptophan is utilized for protein synthesis, tryptophan metabolism could be of special relevance in the elderly. Essential amino acid tryptophan is not only the sole precursor of serotonin and thus important for mood and cognition, but it is also linked to inflammation and immune activation via the so-called kynurenine pathway (KP), which is often systemically upregulated when the immune response is activated [121]. Th1-type cytokine interferon-gamma, among other biochemical pathways, induces tryptophan breakdown by the enzyme indoleamine 2,3-dioxygenase (IDO-1). As a result of the accelerated IDO-1 activity, levels of indoleamines tryptophan and serotonin become diminished, and this may increase the risk of, e.g., cognitive impairments. The activated immune system in older persons can be detected by increased kynurenine to tryptophan concentrations (Fig. 2).

**Figure 3.**
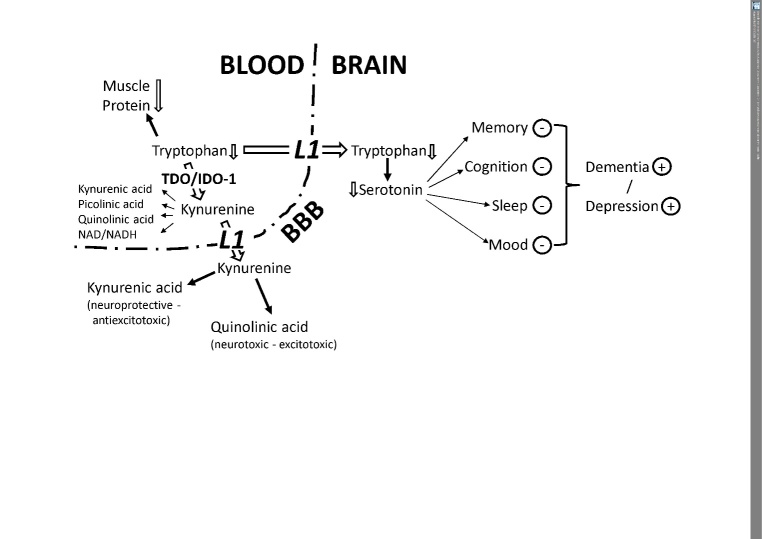
Tryptophan breakdown limits the availability of tryptophan for serotonin synthesis and increases the downstream production of neuroactive metabolites Enhanced tryptophan breakdown by the enzymes tryptophan 2,3-dioxygenase (tryptophan pyrrolase, TDO) and/or indoleamine 2,3-dioxygenase-1 (IDO-1) can affect several body compartments including the brain. Thereby, various intermediate catabolites such as kynurenic acid, picolinic acid, and quinolinic acid are formed on the route to nicotinamide adenine dinucleotides. Tryptophan shortage during/after the pro-inflammatory response may reduce the availability of the essential amino acid for the biosynthesis of muscle proteins and can thus contribute to sarcopenia development with older age. For the transport of tryptophan and kynurenine into the brain to cross the blood-brain barrier (BBB), the leucine-preferring L1 system is utilized in competition with the so-called large neutral amino acids (LNAA). Once arrived in the brain, astrocytes are able to convert kynurenine to neuroprotective kynurenic acid, whereas glial cells primarily produce its neurotoxic counterpart quinolinic acid. Alternatively, tryptophan is converted by the tryptophan 5-monooxygenase to 5-hydroxytryptophan, which decarboxylates to the product serotonin (5-hydroxytryptamin), an important neurotransmitter and precursor of the sleep hormone melatonin. If brain tryptophan is low, serotonin also decreases and can disturb memory and cognition as well as sleep and mood, which finally increase the risk of development of dementia and depression.

Energy restriction clearly results in low plasma tryptophan and hence its availability, which can undermine serotonin metabolism, the KP, and subsequently the immune system [122]. Furthermore, the flux of tryptophan down the hepatic KP is enhanced by competing amino acids such as leucine [123]. Although a high-protein diet provides more tryptophan for the KP, tryptophan availability to the brain is paradoxically decreased as tryptophan competes with the other large neutral amino acids (LNAA) for transport across the blood-brain barrier [124]. For example, a breakfast rich in proteins induces a significant decrease in the plasma total tryptophan to LNAA ratio [125]. Although these changes apply to acute protein intake, a high-protein intake limits tryptophan availability for the cerebral KP, may further influence serotonin synthesis, and can disturb memory and cognition as well as sleep and mood, which eventually increases the risk of development of dementia and depression (Fig. 3).

On the other hand, moderate physical exercise, a potent stimulus to modulate tryptophan metabolism, could be helpful in improving mood status [126]. During exercise, the entry of tryptophan into the brain through the blood-brain barrier is favored by increased muscle use of branched-chain amino acids (BCAAs) and elevated plasma fatty acids. This elevates the ratio of unbound tryptophan to BCAA followed by a substantial increase in tryptophan availability to the brain, consequently leading to higher serotonin concentrations in some areas of the brain [127]. Recent results show that regular endurance exercise also causes adaptations in kynurenine metabolism by increased skeletal muscle kynurenine aminotransferase expression, which shifts kynurenine metabolism away from neurotoxic kynurenine metabolites like quinolinic acid to the production of kynurenic acid. By this method, crossing of kynurenine through the blood-brain barrier and the disruption of neural plasticity are prevented, which can have implications for exercise recommendations for patients with depressive disorders [128].

### Tryptophan-kynurenine, sarcopenia, and longevity

The process of aging is accompanied by chronic immune activation and inflammation, and sarcopenia may represent a consequence of a counter-regulatory strategy of the immune system to dampen the process of immune activation. Thereby, tryptophan breakdown could represent an important checkpoint. Tryptophan deprivation can suppress immune activation processes via restriction of protein biosynthesis and the induction of regulatory T-cells by kynurenine metabolites [129, 130]. Accelerated tryptophan breakdown has been observed in healthy elderly individuals [131, 132]. It may relate to specific clinical diagnoses that are common in old age such as cardiovascular diseases, chronic infections, or cancer. Likewise, not only loss of immunocompetence, but also decline in cognitive abilities and memory and higher risk of depressive mood may develop on the basis of tryptophan deficiency due to accelerated breakdown. Thus, these symptoms may represent side-effects of the immunobiochemical events that derive from chronic immune activation.

For a long time, immunosuppressive and/or anti-inflammatory therapy has been discussed as a prophylactic and therapeutic approach to reduce age-associated ailments and to increase life span [133, 134]. A higher rate of tryptophan breakdown and lower serum tryptophan levels have been described as being associated with a reduced residual life span in individuals with cardiovascular risk, and this was true not only for cardiovascular mortality but also for overall mortality [135, 136]. Interestingly, the immunomodulatory properties of rapamycin and resveratrol are also responsible for their capacity to suppress tryptophan breakdown and IDO-1 activation, which were observed in human peripheral blood mononuclear cells *in vitro* [137, 138]. Whereas intense physical exercise may provoke chronic immune activation and may thus be involved in the development of impaired immune function [139], moderate physical activity and muscular training can be regarded as effective strategies against the overload with antioxidants, but to what extent they might be able to combat aging-associated alterations in tryptophan metabolism remains to be shown.

## Conclusion

Muscular strength represents an independent role in the prevention of chronic diseases, whereas muscular weakness is strongly related to functional limitations and physical disability. Furthermore, low muscular strength has been recognized as an emerging risk factor for premature mortality beyond traditional risk factors such as hypercholesterolemia, obesity, hypertension, and smoking. For the above reasons and because muscle strength is known to decline with age, resistance-type and endurance-type exercise are currently prescribed by numerous health organizations in order to improve fitness and to counteract the adverse effects of aging on health-related parameters, including the risk of morbidity and mortality [140-142]. In addition, the use of dietary protein supplementation can further augment protein anabolism, but can also contribute to a more active lifestyle, thereby supporting well-being and active aging in the older population.
